# Escape Excel: A tool for preventing gene symbol and accession conversion errors

**DOI:** 10.1371/journal.pone.0185207

**Published:** 2017-09-27

**Authors:** Eric A. Welsh, Paul A. Stewart, Brent M. Kuenzi, James A. Eschrich

**Affiliations:** 1 Cancer Informatics, H. Lee Moffitt Cancer Center & Research Institute, Tampa, Florida, United States of America; 2 Department of Thoracic Oncology, H. Lee Moffitt Cancer Center & Research Institute, Tampa, Florida, United States of America; 3 Department of Drug Discovery, H. Lee Moffitt Cancer Center & Research Institute, Tampa, Florida, United States of America; 4 Cancer Biology, University of South Florida, Tampa, Florida, United States of America; The University of Melbourne, AUSTRALIA

## Abstract

**Background:**

Microsoft Excel automatically converts certain gene symbols, database accessions, and other alphanumeric text into dates, scientific notation, and other numerical representations. These conversions lead to subsequent, irreversible, corruption of the imported text. A recent survey of popular genomic literature estimates that one-fifth of all papers with supplementary gene lists suffer from this issue.

**Results:**

Here, we present an open-source tool, Escape Excel, which prevents these erroneous conversions by generating an escaped text file that can be safely imported into Excel. Escape Excel is implemented in a variety of formats (http://www.github.com/pstew/escape_excel), including a command line based Perl script, a Windows-only Excel Add-In, an OS X drag-and-drop application, a simple web-server, and as a Galaxy web environment interface. Test server implementations are accessible as a Galaxy interface (http://apostl.moffitt.org) and simple non-Galaxy web server (http://apostl.moffitt.org:8000/).

**Conclusions:**

Escape Excel detects and escapes a wide variety of problematic text strings so that they are not erroneously converted into other representations upon importation into Excel. Examples of problematic strings include date-like strings, time-like strings, leading zeroes in front of numbers, and long numeric and alphanumeric identifiers that should not be automatically converted into scientific notation. It is hoped that greater awareness of these potential data corruption issues, together with diligent escaping of text files prior to importation into Excel, will help to reduce the amount of Excel-corrupted data in scientific analyses and publications.

## Introduction

Auto-conversion of gene symbols and database accessions by the spreadsheet software Excel (Microsoft, Redmond WA) is a persistent issue in biomedical research. The first published account dates back to 2004, where gene symbols for the Septin family (SEPT1, SEPT2, etc.) and others were reported as automatically converted to date formats within the software (1-Sep, 2-Sep, etc.) [1]. Furthermore, Riken database accessions such as 2310009E13 were reported as converted to the floating-point format of 2.31E+13. A recent programmatic scan of literature [2] revealed that roughly 20% of the manuscripts surveyed are affected by gene symbol auto-conversions in their supplementary material, along with 40% of Excel files deposited in the NCBI Gene Expression Omnibus (GEO) [3]. Additional auto-conversions that occur include subsets of numbers with forward slashes or hyphens (converted to date format), numbers with colons (converted to time format), numbers followed by an A or P (converted to time format), and numbers containing leading zeros (leading zeros are dropped) [4]. Examples of problematic text strings are given in Table 1.

**Table 1:** Escaped vs. unescaped text import into excel.

Escaped	Unescaped
00123456	123456
1234567890123456789	1234567890123450000
2610100E13	26101000000000000000
SEPT7	7-Sep
1/3	3-Jan
1 A	1:00 AM
++ stain intensity	#NAME?

Example strings are given that are auto-converted by Excel when imported from unescaped text files. The escaped text is protected and remains unconverted after import.

In order to avoid unwanted auto-conversions, text files can be imported via the File->Open menu within the Excel program, which then guides the user through options that allow the user to decide which columns to treat as text and which to treat as other data types. However, this method is time consuming, becomes impractical for large numbers of columns, cannot handle columns of mixed data types, and does not allow for customization of any columns beyond the 256^th^ column. Additionally, there is no option to select column data types when a text file is opened via "double clicking", "drag and drop", or "Open With" from the Windows file explorer or desktop. Thus, an automated method of escaping a text file prior to import in Excel, so as to protect vulnerable fields from unwanted auto-conversion, is necessary.

## Methods

According to Microsoft, auto-conversion in Excel can be prevented by manually placing a single quotation (') in front of an entry as an escape character to force the cell to be interpreted as text [4]. However, if this method is used to escape a field within a file prior to importing into Excel, then the resulting field will contain an extra leading single quote after importation that must be dealt with. We have found that escaping a field within double quotes inside of an equation, such as = "*text to escape*", is able to protect strings from auto-conversion on text file import.

Escape Excel scans a tab-delimited text file for entries that need to be escaped. These entries are identified using regular expressions. All fields are first cleaned by removing leading/trailing spaces, enclosing quotes, leading single quotes, and UTF-8 byte order marks. These can interfere with correct parsing of the escaped output field by Excel, as well as interfere with regular expression pattern matching. Entries that are identified as likely to undergo unwanted auto-conversion by Excel are then encapsulated in a text string within an equation (e.g., SEPT1 is escaped as = "SEPT1"). Starting the entry with an equals sign tells Excel to treat the field as an equation, and the quotes tell Excel that the equation consists solely of a string. Once Escape Excel is run on a file, the resulting output can be opened in Excel without auto-conversion issues.

Pseudocode for the algorithm is as follows:

 for each field within a line

  # remove problematic characters

  do

   remove leading UTF-8 byte order mark

   remove pre-existing Escape Excel escaping

   remove enclosing double quotes

   remove remaining leading double quotes

   remove leading spaces

   remove trailing spaces

  while (any cleaning rule was applied)

  # escape numbers

  if field is a number

   if escape leading zeroes flag setting

    escape leading zeroes for integer numeric portion

   end if

   if escape scientific notation flag setting

    escape scientific notation with ≥ 2 digits before decimal (i.e. 12E3)

    escape numbers with > 11 digits before decimal

   end if

  # escape everything else, if paranoid option was set

  else if paranoid flag setting

   escape everything that is not a number

  # escape only potentially problematic fields

  else

   escape leading single quote

   escape leading equals sign

   escape leading +/- if followed by non +/- characters

   if escape dates flag setting

    escape anything that looks like a date and/or time of day

   end if

  end if

 end for

## Implementation

**Fig 1:**
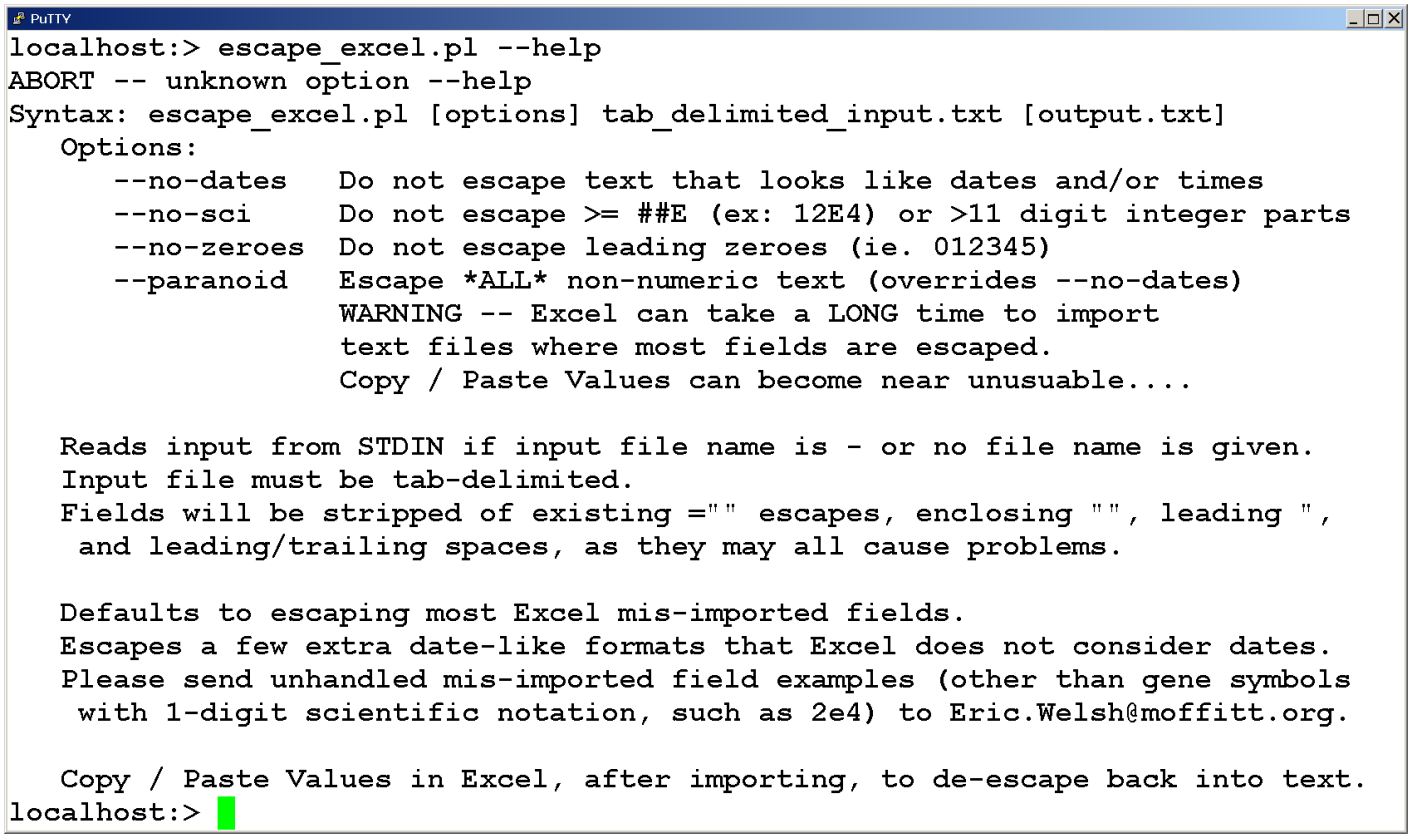
Screen capture of escape excel command line tool help text. Unknown command line options, including—help, will abort the program with a brief usage statement, including command syntax and descriptions of supported option flags.

The original implementation, from which all other implementations derive, is implemented as a Perl script. Thus, the Perl script requires a Perl interpreter to be installed, either on the server, or on the user's local machine, depending on the implementation. Usage information, along with non-default options for disabling certain classes of pattern matching rules, is displayed when the script is run on the command line with the—help option (Fig 1). If difficulties arise when parsing deprecated Mac-style carriage return only end-of-line characters on other platforms, a pipeable eol2eol.c program is included to assist with end-of-line conversions prior to data input. Usage information for eol2eol is displayed when eol2eol is run on the command line with the—help option.

**Fig 2:**
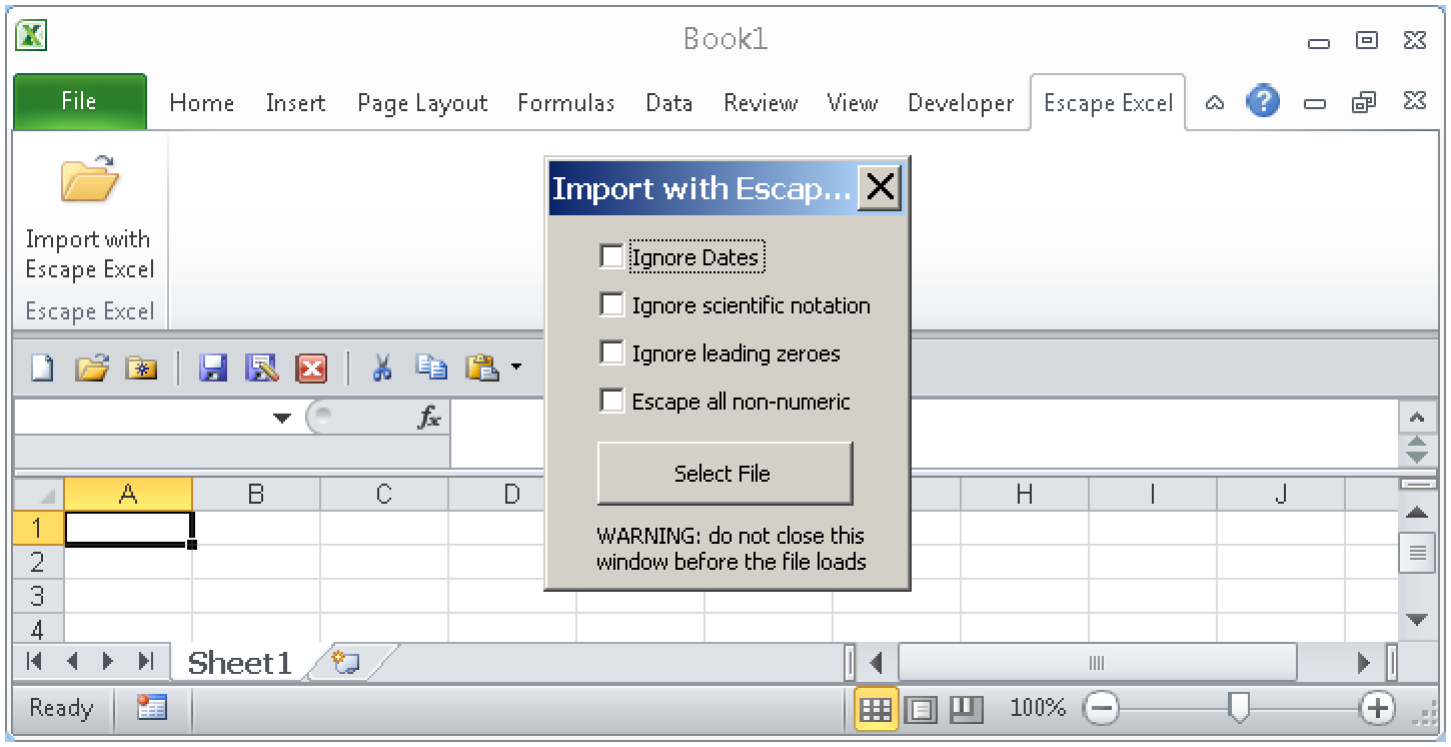
Screen capture of escape excel windows excel Add-In. Installation of the Excel Add-In allows for automatic processing of a selected file with Escape Excel, followed by importation of the escaped file into Excel. The Add-In adds a new Escape Excel tab to the Ribbon, from which non-default options and the file to be imported are selected.

A Windows-only Excel Add-In, which calls the Perl implementation, was implemented in Visual Basic as an.xlam file, and packaged with the InnoSetup installer (http://www.jrsoftware.org/isinfo.php). The Add-In does not require the installation of a Perl interpreter, as the included escape_excel.exe executable consists of a self-extracting archive that runs the script in memory from within an embedded Perl interpreter, packaged with the pp PAR Packer (http://search.cpan.org/dist/PAR-Packer/) using the Strawberry Perl interpreter (http://strawberryperl.com/). The included eol2eol.exe program, called from within the Add-In in order to support carriage return only end-of-line characters, output by default in OS X versions of Excel, was compiled with the i686-pc-mingw32-gcc compiler under Cygwin (https://www.cygwin.com/). The Excel Add-In will only run within Microsoft Windows versions of Microsoft Office, and requires a version of Office supporting the "Ribbon" menu interface (Office 2007 and newer). After installation, the Add-In can be run from the "Import with Escape Excel" icon in the newly-added right-most "Escape Excel" Ribbon tab, after which non-default options can be selected, followed by "Select File" to browse for the file to be imported (Fig 2).

**Fig 3:**
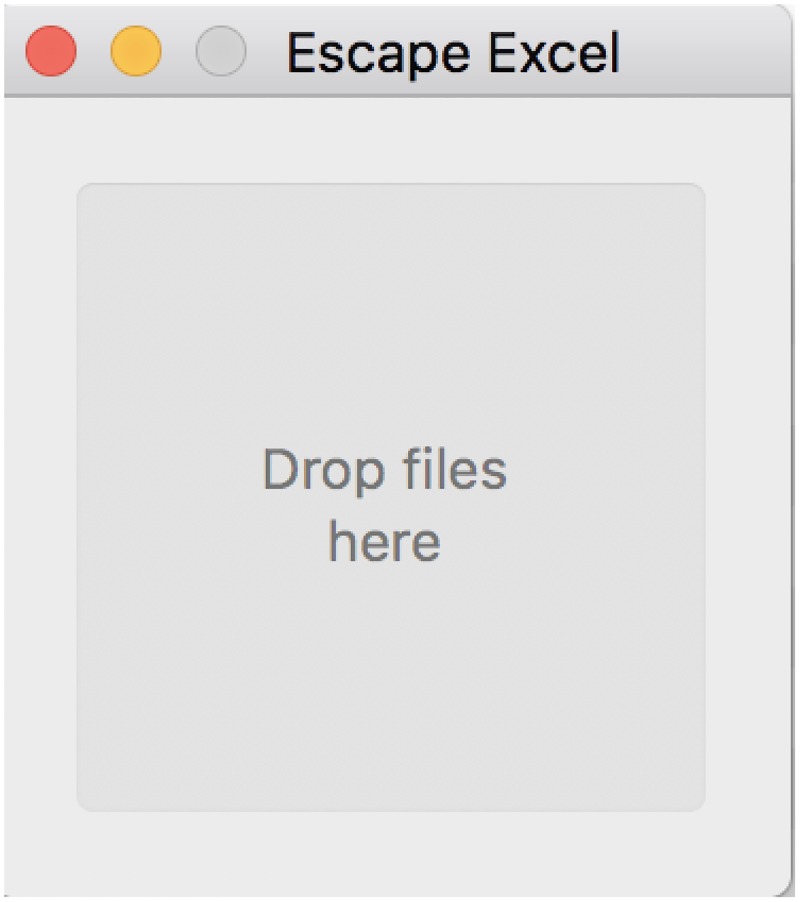
Screen capture of escape excel OS X application. Files to be escaped can be drag-and-dropped onto the application, which will then automatically export escaped versions of the files.

An OS X only application (.app) was implemented by wrapping Escape Excel into an application bundle with an application binary to run the script using Platypus (https://github.com/sveinbjornt/Platypus). Once unzipped, there is no need to install the Escape Excel application, and it will run Escape Excel with default settings upon opening files with the application, or by drag and drop onto the application icon (Fig 3). Non-default options are not supported. Perl is required to run the application, however, Perl is typically packaged with Mac OS X. If needed, Perl can be installed by the user (https://learn.perl.org/installing/osx.html).

**Fig 4:**
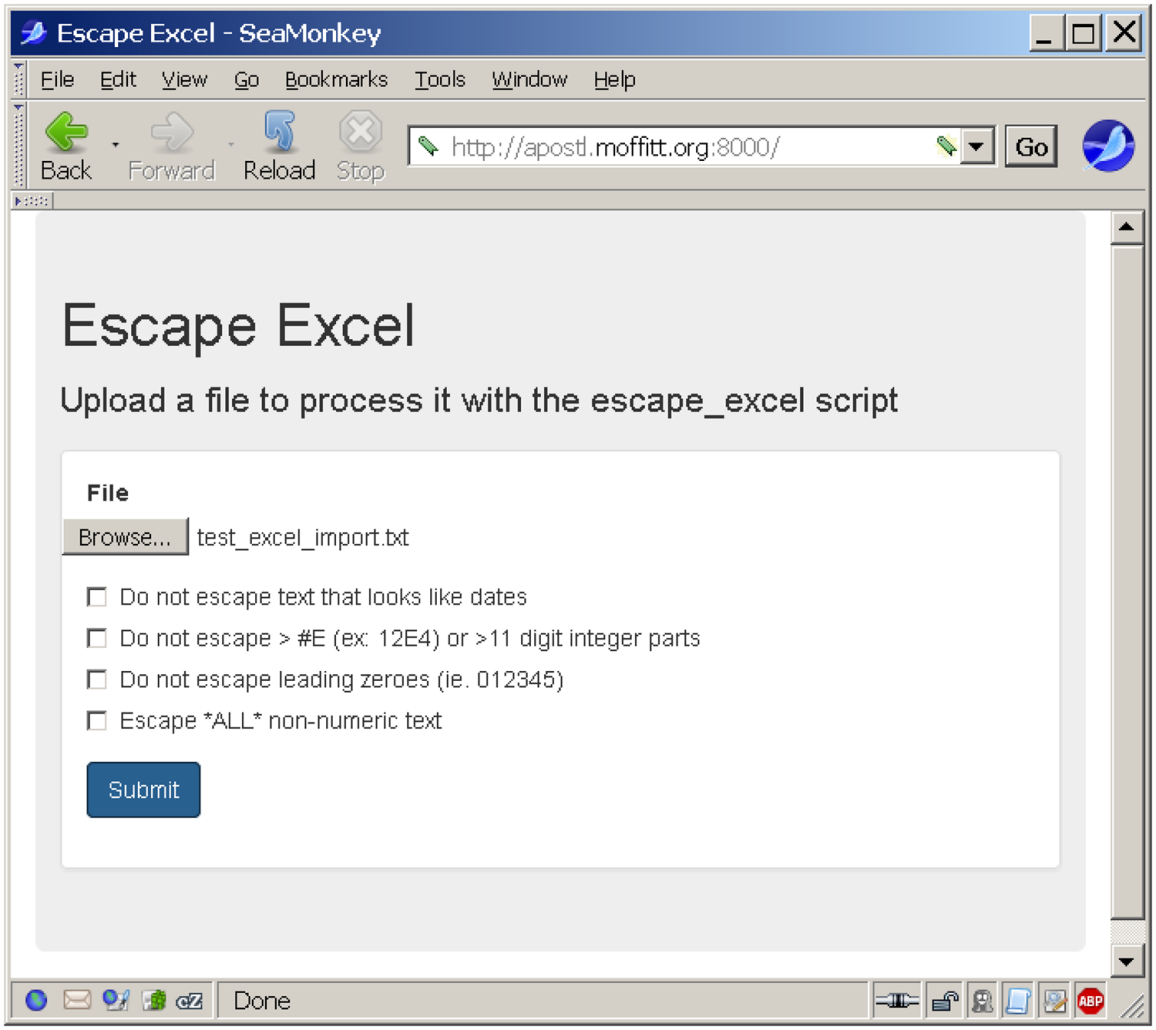
Screen capture of escape excel simple non-Galaxy web server. Non-default options are selected via checkboxes. The file to be escaped is selected via the Browse file requestor. Clicking on the Submit button will prompt the user to download or open the resulting escaped file.

A simple web server was implemented in node.js (https://nodejs.org), using the Express web framework (https://expressjs.com/). The web server displays an HTML form that allows the user to enable the different command-line options via HTML checkboxes (Fig 4). After configuring the options they would like to use, the user can then choose a file from their computer to upload to the server. After the user clicks submit, a POST request is made to /upload, where the file is streamed to the server as multi-part form data. This data is then streamed through escape_excel.pl by spawning a subprocess with the appropriate arguments and streaming the uploaded data through the process. The output of the escape_excel.pl subprocess is then streamed back to the user's web browser—causing their browser to download an escaped copy of the file (with the same name as the original file).

**Fig 5:**
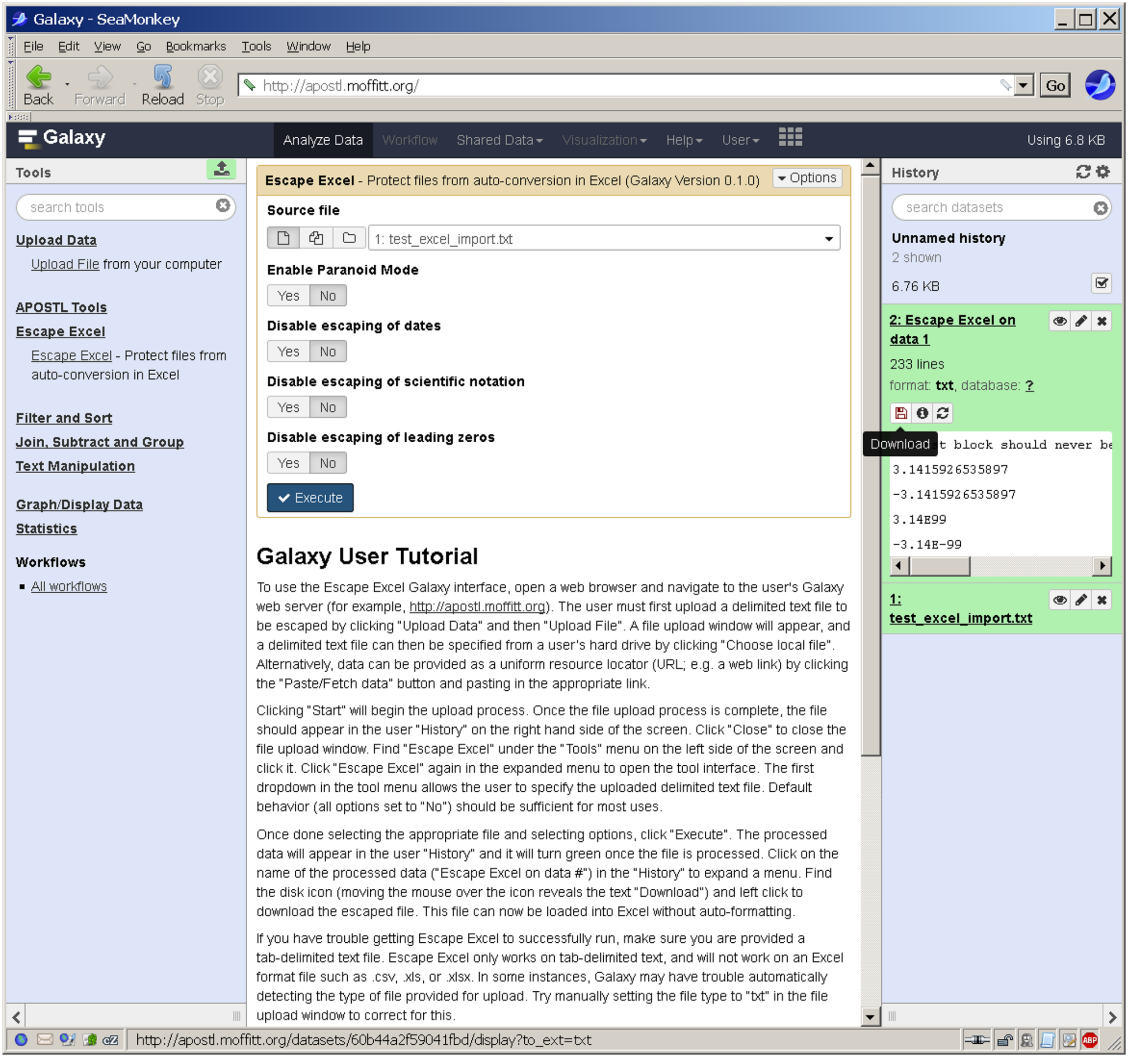
Screen capture of escape excel Galaxy web server interface. The results from an example data processing workflow are shown, ready for download (right pane), after uploading a file with the Upload Data tool (left pane) and processing the selected file with the Escape Excel tool (tool selected in the left pane, options selected in the middle pane). A step-by-step tutorial is provided underneath the form in the middle pane.

The Galaxy interface implementation requires installation under the Galaxy web platform. Galaxy requires UNIX/Linux or Mac OS X with Python 2.7 and git installed. Galaxy can be downloaded from the command prompt by typing "git clone https://github.com/galaxyproject/galaxy.git”. Once cloned, "sh run.sh" will start the Galaxy instance. Galaxy will install any configuration files or Python modules needed. Once started, the Galaxy server can be accessed using a web browser at http://localhost:8080/ by default. Once running, an administrator can install Escape Excel in Galaxy from the Galaxy Tool Shed. Additional Galaxy installation documentation and help can be found at https://galaxyproject.org/admin/get-galaxy/. A detailed usage tutorial is included within the Escape Excel interface, beneath the data processing form (Fig 5). Briefly, the process of escaping a file consists of uploading a file to the galaxy server (Upload Data, top left of the Tool pane), navigating to the Escape Excel tool (left side of the Tool pane) selecting the uploaded Source file to be escaped (top of middle pane), enabling any optional Escape Excel flags, clicking the Execute button, selecting the resulting escaped file from the top right History pane, then clicking on the disk icon beneath the selected results file to download the file.

## Results

We have released the above implementation as Escape Excel, a freely available tool written in Perl for escaping a text file so as to prevent auto-conversion of known problematic entries upon opening in Excel. Escape Excel is implemented as a command line based Perl script, a Windows-only Excel Add-In, an OS X drag-and-drop application, a simple web-server form, and in the Galaxy [5] web environment (see Availability of data and materials). A set of software validation examples of both Excel-safe text strings and strings that are escaped by the software are provided in supplemental S1 Table.

The ability of Escape Excel to prevent the auto-conversion of gene symbols is of especial interest to genomic and transcriptomic data, due to its high prevalence in the scientific literature [1, 2]. Using current official gene symbols for human and mouse (supplemental S2 Table), retrieved from GenBank on March 15^th^ 2017, 34 out of 59726 human genes are auto-converted incorrectly by Excel upon text import, along with 29 out of 68260 mouse genes. Although these problematic gene symbols represent only ~0.05% of all genes, if 5% of all genes were, at random, identified as biologically interesting in a hypothetical experiment, then we would expect, on average, 1–2 human or mouse genes per experiment to be impacted by Excel corruption issues in the results. Although no longer official gene symbols, the deprecated problematic human/mouse aliases 2E4, 2E6, 2E12, 3E2, and 3e46 are left unescaped by Escape Excel, since these text strings could represent normal scientific notation in other contexts. Although all of the problematic human gene symbols, both past and present, are date-related auto-conversions, 37 mouse gene symbols (2 current, 35 aliases) are also impacted by scientific notation auto-conversions, such as 2610100E13, which largely stem from RIKEN identifiers.

## Discussion and conclusions

Some cleanup and alteration of the original input text is performed as part of the pre-processing step of the algorithm. Perl, as well as many other tools that import text files, are not aware of UTF-8 byte order marks, and thus treat them as literal characters. Some products, such as Microsoft SQL Server, export text files with UTF-8 byte order marks, which can lead to string corruption in software that is not aware of them. These superfluous byte order marks are thus removed to prevent potential problems in downstream applications, as well as regular expression matching. Pre-existing escaping from previous passages through Escape Excel, enclosing double quotes, leading double quotes, and leading/trailing spaces are also removed. This process avoids both problems with regular expression matching, as well as potential Excel parsing problems on either the escaped or unescaped output.

The escaping pattern detection rules were all chosen due to Excel corruption issues that we have observed while analyzing various datasets during the course of our research. It is not uncommon, at least within databases at our institution, to use numerical identifiers which contain leading zeroes, zero-padded so as to reach a minimum field width (i.e. 001234). Loss of these leading zeroes during intermediate passages through Excel can result in many failed database queries, as the identifiers are no longer exact string matches to those within the database. Leading equals signs must be escaped to prevent Excel from attempting to evaluate the field as an equation. Excel will also attempt to treat fields such as "++ stain intensity" as an equation, rather than a text string representing the relative amount of staining on a tissue slide, so such fields must also be escaped. Excel will attempt to interpret anything that looks like a time of day (i.e. "1 A", from a series such as "1 A", "1 B", "1 C") into an internal time representation. Escaping has thus been extended to cover combinations of dates and times that Excel is known to treat as dates, so as to preserve original date formats as-is, as well as prevent unwanted conversion of regular text into dates. Leading single quotes must also be escaped to prevent Excel from potentially removing them during text manipulation within Excel.

Escaping of very large whole numbers, as well as less common forms of scientific notation, is required due to common sample/assay identifiers encountered when dealing with various biological data and assays. Escaping of scientific notation with ≥ 2 digits before the decimal point (i.e. 12E3) is necessary for protecting various assays involving plates with sample wells arranged in a grid, where the row identifiers are letters and the column identifiers are numbers. The reported sample identifier from such assays is commonly composed of plate # + row letter + column #. Plate 11, row A, column 9 would result in an identifier of "11A9". This becomes a problem with plates consisting of five or more rows. Plate 11, row E, column 9 would result in "11E9", which Excel would load as the number "1.1E10". Similar problems arise with chip / plate / assay barcodes consisting of long strings of digits, such as 52070900777217100409406332354780 (the barcode of a microarray in one of our internal datasets). Excel automatically converts all whole numbers with ≥ 12 digits into scientific notation, resulting in irreversible identifier corruption unless reformatted to display non-scientific notation with zero decimal places prior to re-exporting to a text file (the reformatting salvaging only works for up to 15 digits of precision, after which all further digits are lost due to Excel's internal floating point representation of numbers).

Most text-related functions in Excel will function properly on the escaped text equations, however, some features, such as Text-to-Columns, will require the escaped text to be copied and pasted as "Paste Values" to convert the equations back into regular text strings before they will work correctly. Although this additional step of converting the escaped text back into regular text is not frequently required, it would be desirable to escape the text in such a manner that the text-within-equations work-around is not required. One such possibility would be to output a Microsoft Office Excel XML file containing the escaped data with problematic fields specified as type "text". Predefinition of the type "text" for each problematic cell would prevent auto-formatting issues imposed by Excel. Although this could be used to import the problematic fields directly as text without going through an equation intermediate, there are several disadvantages to XML file output. The size of the file may increase significantly due to additional XML markup and formatting, which may be problematic for files of several hundred megabytes. The file would also no longer be in the form of a spreadsheet, which may reduce human readability. Although Escape Excel is envisioned to be run just prior to importing the file into Excel, thus obviating the need for compatibility outside of Excel, XML output would decrease compatibility with other software tools, including traditional UNIX row- and column- based command line utilities such as cut, paste, head, and tail, as well as other software that accepts tab-delimited input. We investigated implementation of an XML output version of the software, but encountered compatibility issues between different versions of Office, as well as introducing additional external XML writer library dependencies to the Perl and/or Python runtimes. Due to all of the aforementioned issues with XML output, we have decided to support only text-equation style field protection for now, although we may revisit XML support in future versions of the software.

We have developed and implemented a method for preventing Excel from corrupting imported text files due to unwanted auto-conversions. After processing with the Escape Excel software, text files can be safely imported into Excel without auto-converting gene symbols into dates and other related auto-conversion silent data corruptions. This open source software is freely available, implemented in several different wrappers (command line tool, Windows Excel Add-In, OS X drag-and-drop application, Galaxy interface, and a simple web form for server deployment) for greater user accessibility. While this tool is unable to salvage existing data that has been corrupted by Excel auto-conversion, it is hoped that the availability of this tool will increase awareness of the problem and contribute to reducing its impact on published scientific data sets, analyses, and literature.

## Supporting information

S1 Table Validation set for escape excel. Which fields should, or should not, be escaped are denoted by section header descriptions.
(XLSX)

S2 Table List of human and mouse gene symbols. Symbols were imported as forced-text, unescaped auto-converted fields, and escaped fields. Columns F and G indicate problematic symbols.
(XLSX)

S1 File Implementation of the escape excel algorithm as a Perl script. (PL)

## Abbreviations

BMK: Brent M. Kuenzi
CCSG: Cancer Center Support Grant
EAW: Eric A. Welsh
NCI: National Cancer Institute
PAS: Paul A. Stewart
UTF-8: Universal Transformation Format-8
XML: Extensible Markup Language
